# The Retinoblastoma (RB) Tumor Suppressor: Pushing Back against Genome Instability on Multiple Fronts

**DOI:** 10.3390/ijms18081776

**Published:** 2017-08-16

**Authors:** Renier Vélez-Cruz, David G. Johnson

**Affiliations:** 1 Department of Epigenetics and Molecular Carcinogenesis, The University of Texas MD Anderson Cancer Center, 1808 Park Road 1C, P.O. Box 389, Smithville, TX 78957, USA; 2 Department of Biochemistry, Midwestern University, Chicago College of Osteopathic Medicine, 555 31st Street, Downers Grove, IL 60515, USA

**Keywords:** DNA repair, BRG1, chromatin remodeling, SWI/SNF, E2F1, homologous recombination, EZH2, repetitive sequences, Non-homologous end joining, Suv4-20H

## Abstract

The retinoblastoma (RB) tumor suppressor is known as a master regulator of the cell cycle. RB is mutated or functionally inactivated in the majority of human cancers. This transcriptional regulator exerts its function in cell cycle control through its interaction with the E2F family of transcription factors and with chromatin remodelers and modifiers that contribute to the repression of genes important for cell cycle progression. Over the years, studies have shown that RB participates in multiple processes in addition to cell cycle control. Indeed, RB is known to interact with over 200 different proteins and likely exists in multiple complexes. RB, in some cases, acts through its interaction with E2F1, other members of the pocket protein family (p107 and p130), and/or chromatin remodelers and modifiers. RB is a tumor suppressor with important chromatin regulatory functions that affect genomic stability. These functions include the role of RB in DNA repair, telomere maintenance, chromosome condensation and cohesion, and silencing of repetitive regions. In this review we will discuss recent advances in RB biology related to RB, partner proteins, and their non-transcriptional functions fighting back against genomic instability.

## 1. Introduction

The retinoblastoma (RB) tumor suppressor plays an important role in cell cycle progression [1,2,3]. The function of RB in cell cycle control is mediated through its interaction with the E2F family of transcription factors. RB binds to E2F family members at the promoters of genes important for S phase progression and cell proliferation. The binding of RB to E2F proteins either blocks the recruitment of transcriptional co-activators or recruits transcriptional co-repressors to these promoters, thus repressing the expression of these genes and halting the G1/S cell cycle transition. Upon mitogen stimulation, cyclin-dependent kinases (CDK4, CDK6, and CDK2) become activated and phosphorylate RB [4,5,6]. Hyperphosphorylated RB dissociates from E2F, which allows E2F to recruit transcriptional co-activators to these promoters, thus relieving the transcriptional repression of these genes and allowing cell cycle progression. As cell cycle progresses, the decrease in CDK activity and the activity of protein phosphatase 1 (PP1) dephosphorylate RB (known as hypophosphorylated RB), which forms again a complex with E2F proteins and represses the transcription of cell cycle progression genes [7]. This negative regulation of cell cycle progression is thought to be the main mechanism by which RB suppresses tumor development. RB, however, interacts with more than 200 proteins, many of which are important for multiple processes beyond cell cycle control [8]. Among the proteins and complexes that directly or indirectly interact with RB there are histone acetyltransferases (HATs), deacetylases (HDACs), SWI/SNF chromatin remodelers (SMARCA2, SMARCA4), and DNA repair factors (BRCA1, CtIP, RPA) and many others. Furthermore, RB has been previously described as a “platform for multiple protein contacts” [1] and as a “multi-functional chromatin-associated protein,” not solely a transcriptional repressor for E2F family members [9].

RB is the most-studied member of the pocket protein family, which is composed of RB (p105, *RB1* gene), p107 (*RBL1* gene), and p130 (*RBL2* gene) [1]. Germ line mutations in the *RB1* gene result in retinoblastomas, a rare form of childhood cancer, and also higher risk of osteosarcomas and other types of cancer [10]. Moreover, the majority of human cancers have either mutations in the *RB1* gene, or mutations in other genes in the RB pathway that result in a functionally inactivated RB, such as increased expression of cyclin D, CDK4 or CDK6 or silencing of the CDK inhibitor p16 [11]. It is widely accepted that the negative regulation of cell cycle progression is the main tumor suppressor function of RB. Indeed, studies using mouse models of RB have shown that tumor initiation in the absence of RB requires E2F1, thus supporting the idea that the repression of E2F1 target genes is behind the tumor suppression activity of RB [12]. Both p107 and p130 are also transcriptional regulators that mediate the repression of E2F-target genes by binding to these transcription factors and either blocking the recruitment of transcriptional co-activators or recruiting transcriptional co-repressors to these promoters [1]. However, p107 and p130 are very rarely mutated in human cancers [13]. Furthermore, studies in mice show that RB is an essential gene, as the knock-out mice die during embryonic development [14]. On the other hand, p107 and p130 knock-out mice develop normally, suggesting that p107 and p130 cannot perform all the functions of RB [15,16,17]. While RB is mostly known as a transcriptional repressor with respect to cell cycle control, this tumor suppressor has also been shown to play a role in the transcriptional regulation of genes involved in apoptosis, differentiation, stem cell biology, and cell adhesion [18,19,20,21]. RB also undergoes a number of post-translational modifications in addition to phosphorylation, such as ubiquitylation, SUMOlatyon, methylation, and acetylation [22,23]. Finally, RB performs non-transcriptional cellular functions to maintain genome stability and, with few exceptions, p107 and p130 do not seem to play a role in these functions [24].

The E2F family of transcription factors is the main target of the pocket protein family of transcriptional repressors [25,26]. The E2F family comprises eight members (E2F1-8); E2F1, E2F2, and E2F3 are associated with transcriptional activation and are targets for RB. E2F4 and E2F5 are transcriptional repressors and the targets for p107 and p130, while E2F6, E2F7, and E2F8 are transcriptional repressors independent of RB [25,26]. The best-characterized member of the E2F family is the E2F1 transcription factor, which is often amplified in human cancers (cBioPortal for Cancer Genomics). E2F1 has important roles not only in the transcription of cell cycle regulation genes, but also in the induction of apoptosis [26,27,28]. E2F1 is phosphorylated by the Ataxia telangiectasia mutated (ATM) and ATM Rad3-related (ATR) kinases after the induction of DNA double strand breaks (DSB) and UV damage [29,30]. This phosphorylation site is not conserved in the other members of the E2F family. Importantly, this phosphorylation site is critical for a non-transcriptional function of E2F1 in the repair of these types of DNA lesions, thus like RB, E2F1 has non-transcriptional functions guarding genome stability [28,31,32,33].

Genomic instability refers to a state in which cells accumulate increased levels of genetic changes, which further increases the probability of multiple alterations that could result in tumorogenesis. Genomic instability is a hallmark of cancer and is associated with increased tumor heterogeneity, poor prognosis, and increased risk of therapy resistance [34,35]. The loss, inactivation, or errors in DNA repair pathways, increased replication stress, loss of cell cycle checkpoints, improper chromosome segregation, impaired apoptotic signaling, among others, are some of the challenges that threaten genome stability. Recent work identified new, non-transcriptional roles for RB in maintaining genome stability, which could help explain some of the phenotypes observed in RB-deficient cells and contribute to RB tumor suppressor function. In this review we will discuss recent findings describing new functions for RB in fighting back against genomic instability.

## 2. Retinoblastoma (RB) Role in Double Strand Breaks (DSB) Repair

A plethora of agents and processes constantly challenge the integrity of the genetic material and defects in DNA repair very often result in genomic instability [35,36]. DSBs are among the most toxic and mutagenic types of DNA damage. Blocks to DNA replication, conflicts between transcription and replication machineries, or imbalances in nucleotide pools can induce replication fork arrest or stalling, which could result in DSBs [36]. Moreover, ionizing radiation (IR) and many other forms of cancer treatments kill cells by inducing DSBs [37]. DSBs are repaired mainly by two different pathways in human cells; homologous recombination (HR) and non-homologous end joining (NHEJ) [38,39]. HR uses a sister chromatid as a template to repair DSBs and therefore occurs primarily at late stages of S phase and during the G2 phase of the cell cycle. Because HR uses a sister chromatid as a template, this pathway is thought to be less mutagenic [38]. NHEJ mainly consists of the enzymatic ligation of broken DNA ends and is highly mutagenic [39,40]. Indeed, loss of the HR pathway itself results in increased chromosomal abnormalities and genomic instability due to the overuse of NHEJ [40,41]. HR-deficient cells are also sensitive to chemotherapeutic agents that damage DNA and specifically to poly(ADP-ribose) polymerase inhibitors (PARPi) [37,42,43]. This particular vulnerability is being exploited in the treatment of ovarian cancers with mutations in the breast cancer susceptibility genes (*BRCA1* and *BRCA2*) and other HR-deficient cancers.

**Figure 1:**
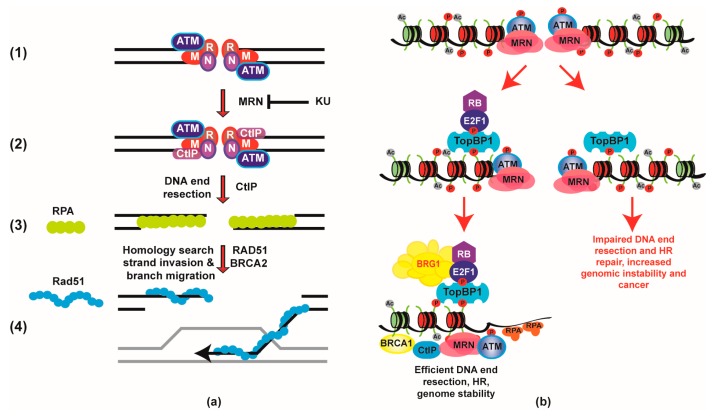
(**a**) The homologous recombination (HR) repair pathway involves multiple steps: (1) double strand breaks (DSB) recognition by the MRE11-RAD50-NBS1 (MRN) complex and the Ataxia telangiectasia mutated (ATM) kinase; (2) the CtIP nuclease is recruited to the DSB to initiate DNA end resection; (3) the replication protein A (RPA) coats the single-stranded DNA (ssDNA) regions and activates the ATM RAD3-related (ATR) kinase; (4) the breast cancer susceptibility gene 2 (BRCA2) protein mediates the replacement of RPA by the RAD51 recombinase, which will catalyze the homology search and the progression of HR; (**b**) RB is recruited to DSB through the TopBP1-E2F1-RB complex, which recruits the BRG1 ATPase. BRG1 stimulates DNA end resection and HR by reducing the nucleosome density at the break site. In the absence of RB, there is no decrease in nucleosome density at the break site, which impairs DNA end resection, HR, and promotes genomic instability.

DSBs are first recognized by the MRE11-RAD50-NBS1 (MRN) complex and the ATM kinase [44]. If the break occurs during the S/G2 phases of the cell cycle and a sister chromatid is available as a template, the break could be repaired through HR. During HR, the DSB undergoes a nuclease-driven process known as DNA end resection in order to generate 3’-end single-stranded DNA (ssDNA) regions that are important for homology search and strand invasion later during the recombination process (Figure 1a). DNA end resection is initiated by the MRE11 nuclease within the MRN complex together with the CtIP nuclease [45]. These ssDNA regions are coated by RPA and this RPA-coated structure recruits and activates the ATR kinase, which in turn activates the Chk1 kinase [46]. This repair pathway is known to require chromatin remodelers, modifiers, and even the incorporation of histone variants in order to deal with the barrier that nucleosomes pose to the resection machinery [47,48].

We recently showed that RB is recruited to DSBs and that RB-deficient cells display a defect in DNA end resection and HR [32]. These DNA repair defects were also observed in E2F1-deficient cells [32,49]. We identified a novel complex containing TopBP1-E2F1-RB-BRG1, which recruits BRG1 to DSBs and stimulates DNA end resection likely by decreasing the nucleosome density at the site of the break (Figure 1b). The formation of this complex requires the phosphorylation of E2F1 on serine 31 (serine 29 in mouse E2F1) by the ATM kinase [29,32]. This phosphorylated form of E2F1 is recognized by the TopBP1 protein, which in turn recruits E2F1 to DSBs [32,50]. TopBP1 is a DNA repair protein important for the activation of the ATR kinase and DNA replication. TopBP1 contains nine BRCA1 C terminal (BRCT) domains that interact with a variety of phosphorylated proteins [50,51]. The sixth BRCT domain of TopBP1 interacts with phosphorylated E2F1 [32,50,51,52]. RB plays a stabilizing role within this complex by shielding phosphorylated E2F1 from proteasomal degradation, as previously observed under different conditions [32,53,54,55]. RB-deficient cells display increased levels and slower clearance of γH2AX nuclear foci, consistent with a DNA repair defect. RB-deficient cells also display decreased levels of DNA end resection and ATR activation, increased levels of chromosomal abnormalities after IR, and sensitivity to chemotherapeutic agents including PARPi, all of which is consistent with a defect in HR. We observed the same DNA repair defects in mouse embryonic fibroblasts (MEFs) from the *E2f1^S29A/S29A^* knock-in mouse model developed by our laboratory [33]. Moreover, the *E2f1^S29A/S29A^* mice are sensitive to IR, indicating that this repair function for RB and E2F1 has real physiological consequences in mice, given that transcriptome analysis showed only subtle transcriptional differences between wild type and *E2f1^S29A/S29A^* MEFs [32,33].

This novel function for RB in HR represents an important extension of previous work from our laboratory showing that E2F1 plays a role in the repair of DSBs [49]. Moreover, it raises new questions as we have also shown that either the absence of E2F1 or the presence of the *E2f1^S29A/S29A^* mutation results in a defect in the repair of UV-induced DNA damage and the *E2f1^S29A/S29A^* mouse is susceptible to UV-induced skin cancer [33,56,57]. Since both UV and IR induce the phosphorylation of E2F1 on serine 31, it would be of interest to determine whether RB also stabilizes phosphorylated E2F1 after UV, as it does after IR [32]. A few studies have shown a potential role for RB in the repair of UV damage through either the transcription or stabilization of repair factors, but clear mechanistic study addressing this question would be of interest [58,59]. There are differences and similarities in the mechanism of E2F1 in the repair of UV lesions and DSBs. E2F1 stimulates the repair of UV photoproducts through the recruitment of the GCN5 HAT to sites of damage, which increases histone H3 lysine 9 acetylation (H3K9ac) and enhances the recruitment of the nucleotide excision repair (NER) factors to the damage sites [33,56,57]. In the case of DSB, E2F1, together with RB, recruits the SWI/SNF BRG1 ATPase to DSBs and BRG1 remodels chromatin at the break site, thus allowing the initiation of DNA end resection [32]. Recent studies have shown that BRG1 is important for DNA end resection in yeast [60] and that the removal of nucleosomes during DNA end resection is likely important for the efficiency of the process [61]. It is important to note that in a way, RB and E2F1 are using their “canonical functions,” since both of these factors interact with these chromatin remodelers or modifiers in the context of transcription [62,63]. Independently of the repair pathway, RB and E2F1 are being shared by the transcription and repair machineries to capitalize on the capacity of these factors to recruit chromatin modifiers and remodelers to damage sites and thus stimulate the repair process [31,64]. It may seem counterintuitive to think of RB as a repressor and yet opening up chromatin to help the repair process, but it is important to remember that both RB and BRG1 have been shown to participate in transcriptional activation and repression [18,20]. This mechanism of recruitment of chromatin remodelers and modifiers to DSBs is just one, likely among many others. These chromatin remodelers and modifiers can also be recruited through a variety of additional interactions with other repair proteins to damage sites as well.

The NHEJ pathway repairs the majority of DSBs in human cells as it is faster, albeit less accurate, than HR [39,40]. During NHEJ, the DSBs are recognized by the KU70–KU80 heterodimer, followed by the stabilization of the NHEJ machinery at the break site, the bridging of the DNA ends, activation of the DNA-dependent protein kinase (DNA-PK), end processing (if needed), and finally the ligation of the DNA ends by DNA ligase 4 [65]. A proteomic analysis showed that RB, p107, and p130 interact with both KU70 and KU80 and the inactivation of either RB or the three members of the pocket protein family resulted in a NHEJ defect [66]. This study also showed that the inactivation of RB results in increased chromosomal abnormalities and higher levels of γH2AX after IR. The authors proposed that the physical interaction between the N terminal of RB (or the other pocket protein family members) is important for efficient NHEJ. Whether RB is recruiting a chromatin remodeler or modifier to break sites in the case of NHEJ is unknown and should be addressed in the future. It is possible that the role RB plays in NHEJ may be a purely physical one, as a platform for protein–protein interactions. While there are multiple reports of chromatin remodelers and modifiers affecting NHEJ [67,68,69], there is likely much more need for chromatin remodeling during HR because the DNA ends need to be processed by nucleases and the nucleosome impedes such processing [47,61,70]. Future studies should address how exactly RB is contributing to NHEJ.

It seems that RB contributes to both DSB repair pathways, HR and NHEJ [32,66]. RB is thought as “active” (hypophosphorylated RB) and associated to E2F1 during G1, the phase when NHEJ would take place. E2F1 was not, however, detected in the RB-KU70-KU80 complex likely due to the fact that the authors used an N terminal fragment of RB for their interaction studies [66]. This raises the question of whether E2F1 would play a role in NHEJ. There are two important points to take into account regarding the phosphorylation state of RB at a given phase of the cell cycle; first, this state could change quickly upon DNA damage; second, it is likely that there are several pools of RB that could undergo different post-translational modifications and exist within different complexes simultaneously [23]. Indeed, we observed a strong association between E2F1 and RB after IR [32]. Future studies should address which form of RB and E2F1 are mediating these non-canonical functions for these transcription factors, also taking into consideration that in addition to phosphorylation, these proteins are also acetylated and methylated upon DNA damage [71,72,73,74,75].

## 3. RB Role Silencing Repetitive Sequences

Repetitive DNA sequences comprise a large part of the genomic material (approximately 50% in higher eukaryotes) [76]. These repetitive sequences can be found at satellite DNA (i.e., centromeric and pericentromeric regions), transposable elements, and telomeres [77,78]. Because of where these sequences are found and their structural role in these regions, these sequences must be maintained transcriptionally repressed through a heterochromatic state [78]. Histone modifications and DNA methylation are the mechanisms by which these regions are kept repressed. These regions are characterized by overall hypoacetylation of histones and an increase in repressive marks such as H3K9me3, H4K20me3, and H3K27me3, which are required for the maintenance of these structures [77,78,79]. Loss of this heterochromatic state can result in chromosomal segregation defects, chromosome instability (CIN), illegitimate recombination events, and transposable element-induced genetic alterations [79].

Recently, RB was identified as an important partner of the EZH2 methyltransferase during H3K27me3 deposition in repetitive sequences [80]. ChIP-seq analysis showed that RB was localized to a large extent to repetitive regions and its localization to these regions requires E2F1. The authors found that the majority of RB and E2F1 was bound to intronic and intergenic regions (>95%) and specifically to multiple types of transposable elements, such as short interspersed nucleotide elements (SINE), long terminal retroviruses (LTR), and long-interspersed nucleotide elements (LINE) [80]. The localization of RB and E2F1 to these regions was enriched in arrested cells, but also occurred in proliferating cells. Ishak et al. used a mouse model with an RB mutation (F832A, termed *Rb1^S^*) that impaired the specific interaction between E2F1 and the C terminal of RB [80]. This particular interaction between E2F1 and RB does not occur through the “pocket domains” of RB and is thought of as “non-canonical.” This interaction is also resistant to RB hyperphosphorylation and thus it is thought not to play a role in cell cycle regulation [81,82]. Both *Rb1^S/S^* and *Rb1^−/−^* MEFs showed loss of EZH2 recruitment and H3K27me3 at repetitive sequences and failed to silence these repetitive regions [80]. On the other hand, H3K9me3 and H4K20me3 distribution on repetitive regions was unaffected in *Rb1^S/S^* MEFs, even though a modest increase in H3K9 acetylation of repetitive regions was observed in these cells. In order to assess the physiological consequences of this novel RB function, the authors aged *Rb1^S/S^* mice and discovered that these mice had shorter cancer-free survival and developed lymphomas. Moreover, analysis of these tumors in *Rb1^S/S^* mice showed increased expression levels of these repetitive regions, thus suggesting that this repetitive region silencing function is important for the tumor suppressor activity of RB [80].

It is interesting that this novel RB function in silencing repetitive sequences also depends on E2F1. It would be of interest to determine in the future how does E2F1 (or RB) recognize these repetitive regions. Of note, while the *Rb1^S/S^* mice displayed this defect, mutations of other important domains of RB had no effect on this function; mutation of the domain that interacts with the transactivation domain of E2F proteins had no effect in the silencing of this region and neither did mutation of the LXCXE domain, which interacts with multiple transcriptional regulators. This mechanism for recruitment of EZH2 to repetitive regions may not be the only one. The repression of repetitive regions is very important during development and defects usually prevent embryonic development before the implantation stage, which is not the case for *Rb1^S/S^* or *Rb1^−/−^* mice [14]. On the other hand, the fact that *Rb1^S/S^* mice develop lymphomas resembles the phenotype of *E2f1^−/−^* mice, which develop lymphomas at a similar age [83].

## 4. RB Role in Telomere Maintenance

Telomeres are complex structures at the end of chromosomes that are composed of long stretches of repetitive DNA with a particular chromatin structure and enveloped by a specialized group of proteins known as the shelterin complex. This complex protects telomeres and avoids chromosome ends being mistaken for a broken DNA end [84]. Telomeres are extended by the telomerase complex and the absence of telomerase causes progressive attrition of chromosome ends finally resulting in the loss of genetic material, chromosomal fusions and translocations, and replicative senescence and/or cell death [84]. Moreover, a defect in any of the sheltering components or changes in chromatin structure at the telomeres could result in faster telomere attrition, which eventually will lead to chromosome end-to-end fusion, resulting in dicentric chromosomes, missegregation defects and genomic instability [84].

Multiple studies have linked RB to telomere biology [85,86,87,88]. One study linked RB and the pocket protein family members to telomere length [85]. This study showed that genetic inactivation of p107 and p130 (DKO, double knock-out cells) or RB, p107, and p130 (TKO, triple knock-out cells) resulted in elongated telomeres [85]. This study unfortunately did not provide a mechanistic explanation for the elongation of telomeres in the DKO and TKO cells, but it showed that RB was not the main contributor since *Rb1^−/−^* cells showed normal length telomeres. This is one example in which all members of the pocket protein family seem to have overlapping roles and to functionally compensate for each other to control a specific function.

Another study from the same group showed that TKO cells display a defect in global heterochromatin maintenance specifically at telomeres and centromeres, and a centromere structure defect [86,88]. The authors showed that the global levels of H4K20me3 and DNA methylation were reduced in TKO cells [86]. This was accompanied by an increase in the global levels of histone H3 acetylation. Histone H4K20me3 is particularly enriched at centromeres and telomeres, but this enrichment was not observed in TKO cells. None of the single knock-out cells displayed this defect, indicating that there is functional compensation between pocket protein family members in this respect. Moreover, the authors used an E2F1 mutant that does not interact with RB (E2F1-DB, which has an E2F1 transactivation domain deletion) and showed that expression of E2F1-DB in wild type cells did not result in these heterochromatin formation defects. It is worth noting that the E2F1-DB mutant is thought of mostly as a dominant negative for the transcriptional function of RB, as it would compete out E2F1 transcriptional binding sites, but it is unclear what effect (if any) it would have with respect to non-transcriptional functions of RB and E2F1. Finally, this study showed that the RB protein family interacts with Suv4-20h1 and, to a lesser extent, with Suv4-20h2, the enzymes that methylate H4K20 but that RB was not required for the recruitment of these enzymes to the repetitive regions. The authors proposed that the RB family is important for the maintenance of global heterochromatin structure, including telomeres and centromeres, by somehow stabilizing H4K20me3. This defect with lower H4K20me3 in TKO cells could potentially explain the abnormally long telomeres in these cells [85,88].

Telomeres represent a protective mechanism from the dangers of genomic instability that arise due to the sensitive nature of chromosome ends [84,89]. A defect in telomere maintenance or stability could have dire consequences in genome stability and could contribute to the tumor suppressor function of RB.

## 5. RB Role in Centromere Structure and Chromosome Instability (CIN)

Centromeres are structures on the chromosomes where sister chromatids and spindle apparatus attach. Centromeres consist of repetitive sequences where condensin proteins can be found and possess a heterochromatic structure [90]. Defects in centromere structure can lead to poor sister chromatid attachment, which could result in chromosome missegregation and aneuploidy, or chromosomal breakage and chromosomal loss, known as chromosomal instability (CIN). CIN is a form of genomic instability and is a characteristic of many cancers with poor prognosis and likely to develop therapy resistance [91].

A number of studies linked RB to centromere structure and CIN [88,92,93]. Gonzalo et al. described “butterfly chromosomes” and aberrant centromeres and linked them to a defect in H4K20me3 at centromeres and telomeres in TKO MEFs [86,88]. Later, Manning et al. did a careful analysis of centromere function in RPE-1 cells knocked down for RB [94]. This study showed that the loss of RB caused frequent missegregation of whole chromosomes [94]. This phenotype was caused by an underlying defect in centromere structure that decreased centromere rigidity and did not allow for proper kinetochore-microtubule attachment. The basis for this defect was a premature loss of sister chromatid cohesion in RB-depleted cells. Performing an elegant set of experiments, the authors determined that this centromere problem stemmed from a defective loading of the condensin II complex (CAP-D3) onto chromatin in the absence of RB. Drosophila RB (RBF1) had been shown to interact with the condensin II protein dCAP-D3 through the LXCXE domain of RB and promote its association with chromatin [95]. Manning et al. noted that the inactivation of RB is “a subtle enemy during tumorogenesis because it reduces the fidelity of mitosis without causing more dramatic changes that would compromise cell proliferation” [94]. This is an important consideration, not only for this function of RB, but also for other functions in repair as well. Subtle defects in these functions guarding genome stability allow the cells to propagate with an increasing number of genetic changes, some of which could render the cell malignant and/or resistant to therapy.

In a different study, RB-depleted cells were also found to have a problem in chromatin compaction, in addition to the chromosome cohesion [96]. This defect in chromatin compaction and cohesion was explained by lower levels of chromatin-bound cohesin proteins (SMC1/3). Since there is not a link between cohesin-loading and RB, the authors explored whether chromatin modifications could affect the enrichment of cohesins on pericentromeric regions. This study [96], in agreement with a previous study [86], showed that the deposition of H4K20me3 was important for restoring normal levels of chromatin-bound cohesin and reversing the chromatin-compaction and cohesion defect. Importantly, this study showed that over expression of Suv4-20h2 could correct the chromatin compaction, cohesion, and chromosome segregation problem observed in the absence of RB [96]. The finding that H4K20me3 is the main cause of CIN in the absence of RB could potentially open therapeutic opportunities to reverse or decrease CIN in certain cancers, given that RB is lost at a high frequency in many cancer types. This study also found that the absence of RB caused problems such as increased DNA damage and slow and stressed replication forks, which could also result in genomic instability and has been reported in multiple studies [32,66,96,97].

The importance of the role of RB in CIN through its control of chromatin cohesion and compaction has also been studied in vivo using mouse models. Coschi et al. took advantage of the reported interaction between the LXCXE domain of RB and the condensin II subunit CAP-D3 to test whether the impairment of this interaction contributed to tumorogenesis and genomic instability [98,99]. The authors used a previously developed mouse model with the mutated RB LXCXE domain (known as *Rb1*^Δ*L/*^^Δ*L*^) [99]. This domain is important for the interaction between RB and many chromatin-interacting proteins, remodelers, and modifiers including CAP-D3 [95,99]. Coschi et al. showed that *Rb1*^Δ*L/*^^Δ*L*^ cells display a centromere defect and that this defect was unrelated to the cell cycle control effects that the absence of RB could cause since they were present in *Rb1*^Δ*L/*^^Δ*L*^ mouse embryonic stem cells (mESC, which lack the capacity to arrest cell cycle at G1) and MEFs (which can be arrested in G1) [98]. *Rb1*^Δ*L/*^^Δ*L*^ cells also showed a chromosome condensation delay and reduced levels of condensin II on chromatin when compared to wild type cells. More importantly, the authors tried to determine the effect that this chromosome segregation defect would have in tumorogenesis by crossing *Rb1*^Δ*L/*^^Δ*L*^ mice with *Trp53^−/−^* mice. Since the *Rb1*^Δ*L*^ mutation and *Trp53* deletion both impair G1 arrest after DNA damage; a comparison between *Rb1*^Δ*L/*^^Δ*L*^; *Trp53^−/−^* vs. *Trp53^−/−^* mice would interrogate the contribution of the mitotic function of RB to tumorogenesis [100,101]. The authors found that *Rb1*^Δ*L/*^^Δ*L*^; *Trp53^−/−^* mice had a shortened tumor-free survival when compared to *Trp53^−/−^* mice [98]. More importantly, they also found that the tumors of *Rb1*^Δ*L/*^^Δ*L*^; *Trp53^−/−^* mice were more aggressive than those of *Trp53^−/−^* mice. Finally, in order to test the effect that the *Rb1*^Δ*L*^ mutation may have in genomic instability, the authors crossed *Rb1*^Δ*L/*^^Δ*L*^ with *Trp53^+/−^* mice. Since *Trp53^+/−^* mice develop a similar spectrum of tumors to *Trp53^−/−^* mice and the limiting step is thought to be the loss of heterozygosity event of the wild type *Trp53* allele, these mice have been used as a measure of genomic instability. The authors found that *Rb1*^Δ*L/*^^Δ*L*^; *Trp53^+/−^* mice had a shorter tumor-free survival than *Trp53^+/−^* mice and that these tumors had lost the wild type *Trp53* allele, thus indicating that the *Rb1*^Δ*L/*^^Δ*L*^ mutation results in genomic instability.

## 6. Conclusions

**Figure 2:**
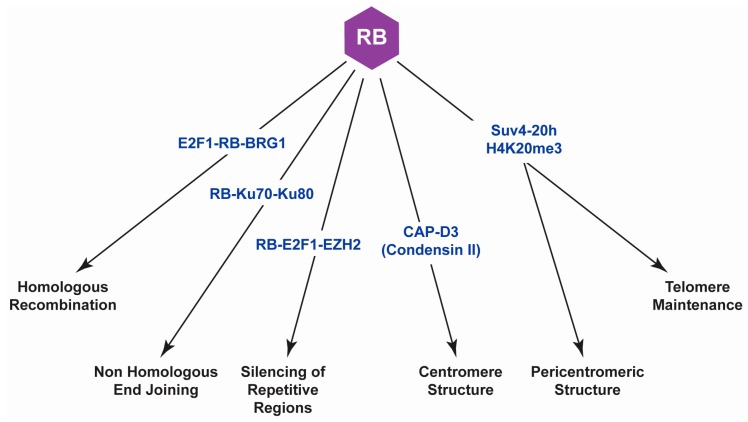
Retinoblastoma (RB) plays important roles in multiple processes unrelated to cell cycle control which, when defective, promote genomic instability.

RB was the first tumor suppressor identified and is mostly known for its central role as a negative regulator of cell cycle progression [1,10,34]. While it is clear that the role RB plays in cell cycle control is important for its tumor suppressor function, studies have found that this tumor suppressor and its multiple protein partners are also involved in many other cellular processes that could also contribute to tumor suppression [9,88,92,102]. Many of these non-canonical functions ascribed to RB are related to genomic instability, a hallmark of cancer associated with poor prognosis, tumor heterogeneity and the development of therapy resistance [35,103]. We have discussed here a variety of non-canonical functions for RB in DNA repair, chromosome condensation and cohesion, centromere and telomere structure, and the silencing of transposable elements (Figure 2). There are also a number of other functions, albeit transcriptional in nature, that are unrelated to cell cycle control and have been described elsewhere [18,19,20,104,105,106]. The uncovering of these non-canonical functions is of critical importance because they cannot only help us understand how RB deficiency results in tumorogenesis, but they could also help us develop therapies against retinoblastomas and other RB-deficient cancers.

The inactivation of RB in cancers can occur in multiple ways. First, genetic somatic alterations of the *RB1* gene can occur and germ line mutations result in retinoblastomas. But the RB pathway itself can be inactivated in various ways as well, for instance, over expression of the cyclin D, CDK4, or CDK6, all of which phosphorylate and “inactivate” RB [4,5,107]. Furthermore, the silencing or deletion of the CDK inhibitor p16 also results in a functional inactivation of the RB pathway [11,108]. Finally, there is also the inactivation of the RB pathway by interaction with viral proteins [109]. How all these different mechanisms of deregulating the RB pathway affect the multiple non-canonical functions of RB in DNA repair, silencing of repetitive regions, centromere and chromosome structure, and others is currently unknown and should be investigated in the future.

We should not assume that the phosphorylation of RB would *de facto* mean that the protein is inactivated, especially in some cases where RB is thought of as playing a structural role as a protein platform mediating protein interactions. Given that RB undergoes multiple post-translational modifications upon different stimuli and it seems to be part of multiple protein complexes, special attention should be paid to the particular form of RB contained within different complexes. For instance, the TopBP1-E2F1-RB-BRG1 complex that we identified as important for HR contains phosphorylated E2F1 on serine 31, but we do not know currently which form of RB is contained within this complex [32]. RB is phosphorylated, methylated, and acetylated after DNA damage and it is therefore critical for us to understand whether particular post-translational modification may be important for specific functions [4,22,74,110,111].

The E2F1 transcription factor is one of the main partners of RB and important for some of the non-canonical functions of RB [32,80]. Not every study that identifies a novel, non-canonical function for RB addresses whether E2F1 also plays a role in such functions. Given the strong and continuous interaction between RB and E2F1, it should not be surprising that E2F1 co-operates with RB in multiple functions. The knock-out mouse models, however, show that RB has essential functions during development, while E2F1 does not [14,15]. It is possible that some of the eight other members of the E2F family could compensate for the absence of E2F1 in some instances. Future studies should always keep in mind the potential contribution of E2F1 to any potential RB function. E2F1 also undergoes multiple post-translational modification such as phosphorylation, methylation and acetylation and, with few exceptions, it is not known how these modifications affect E2F1 non-canonical functions [32,33,49,56,57,112]. Similarly, the other members of the pocket protein family should be considered while addressing novel functions for RB. While these proteins clearly cannot compensate functionally for the loss of RB during development, there are other functions that these proteins may be able to perform. For example, many of the defects in centromere structure and telomere maintenance were first observed in TKO cells and there was no observable defects in single knock-out cell lines [66,85,86,97].

Finally, it is very important to notice the subtleties of the defects observed in RB-deficient cells and the potential consequences that such modest defects in multiple processes could have on cellular transformation. In some cases, the subtle defects observed in these studies may be due to the imperfect system we employ by using shRNAs, which fail to deplete the protein of interest completely. This problem will soon be overcome with the advent of CRISPR/Cas9 technologies for gene editing. In other cases the small defects could arise due to compensating functions by other pocket protein family members. In any case, small or subtle defects are important because they can allow a cell to continue to propagate and accumulate genetic changes or lesions that could contribute to therapy resistance, for instance. It is also important to recognize that these non-canonical functions of RB may only constitute *one* mechanism for such a function among many. For instance, in the case of RB recruiting BRG1 to DSBs it is possible that there are other mechanisms by which this SWI/SNF complex may be recruited to DSBs. Indeed, other subunits of this complex interact with repair factors and those interactions are reported to be important for SWI/SNF recruitment to DSBs [113,114]. It is also possible that not every break requires chromatin remodeling to the same extent, and that may be why the defect is somewhat subtle. Another example is the silencing of repetitive sequences, a process that is absolutely critical during development at the pre-implantation stage. RB knock-out mice develop past this point, thus suggesting that the silencing of these regions occurs through another mechanism during development.

With the development of more sensitive methods to identify particular complexes and the post-translational modifications of the components of such complexes, these and more functions of RB in specific processes will be identified. Similarly, the development of imaging methods will allow us to identify the localization of modified RB and how that may change upon stimuli. All this information will help us build a better picture detailing the etiology of retinoblastomas and other RB-deficient cancers and allow us to develop new therapies.

## Abbreviations

RB	Retinoblastoma
CDK	Cyclin-dependent kinases
PP1	Protein phosphatase 1
HAT	histone acetyltransferases
HDAC	Histone deacetylase
BRCA1	Breast cancer susceptibility gene 1
RPA	Replication protein A
ATM	Ataxia telangiectasia mutated kinase
ATR	Ataxia telangiectasia RAD3-related kinase
DSB	DNA double strand breaks
IR	Ionizing radiation
HR	Homologous recombination
NHEJ	Non-homologous end joining
PARPi	Poly (ADP-ribose) polymerase inhibitors
MRN	MRE11-RAD50-NBS1 complex
ssDNA	Single-stranded DNA
Chk1	Checkpoint kinase 1
BRCT	BRCA1 C terminal domain
MEF	Mouse embryonic fibroblasts
NER	Nucleotide excision repair
DNA-PK	DNA protein kinase
DKO	Double knock-out (p107^−/−^; p130^−/−^)
TKO	Triple knock-out (RB^−/−^;p107^−/−^; p130^−/−^)
CIN	Chromosome instability
